# Epidemiology and antimicrobial resistance of methicillin-resistant *Staphylococcus aureus* isolates colonizing pigs with different exposure to antibiotics

**DOI:** 10.1371/journal.pone.0225497

**Published:** 2019-11-20

**Authors:** Elizeth Lopes, Teresa Conceição, Laurent Poirel, Hermínia de Lencastre, Marta Aires-de-Sousa

**Affiliations:** 1 Laboratory of Molecular Genetics, Instituto de Tecnologia Química e Biológica António Xavier, Universidade Nova de Lisboa, Oeiras, Portugal; 2 Emerging Antibiotic Resistance Unit, Medical and Molecular Microbiology, Department of Medicine, University of Fribourg, Fribourg, Switzerland; 3 French INSERM European Unit, University of Fribourg (LEA-IAME), Fribourg, Switzerland; 4 National Reference Center for Emerging Antibiotic Resistance, Fribourg, Switzerland; 5 Laboratory of Microbiology and Infectious Diseases, The Rockefeller University, New York, New York, United States of America; 6 Escola Superior de Saúde da Cruz Vermelha Portuguesa, Lisbon, Portugal; Kent State University, UNITED STATES

## Abstract

**Background:**

In 2016, very high rates of methicillin-resistant *Staphylococcus aureus* (MRSA)-ST398 (99%) were found in Portuguese pig farms that used colistin, amoxicillin, and zinc oxide as feed additives. Since then, farms A and B banned the use of colistin, and farm C banned the use of both antibiotics.

**Objective:**

The aim of the present study was to evaluate the impact of the ban of colistin and amoxicillin on pig MRSA carriage rates, clonal types and antimicrobial resistance, compared to the results obtained in 2016.

**Methods:**

In 2018, 103 pigs (52 from farm B using amoxicillin only as a feed additive and 51 from farm C where no antibiotics were included in the feed regimen) were nasally swabbed for MRSA colonization. Isolates were tested for antimicrobial susceptibility, and characterised by *spa* typing, SCC*mec* typing and MLST. Whole genome sequencing (WGS) was performed for representative isolates.

**Results:**

Overall, 96% of the pigs swabbed in 2018 carried MRSA, mostly ST398-SCC*mec* V-*spa* types t011/t108. MRSA from pigs not receiving antibiotics in the feed regimen showed susceptibility to a higher number of antibiotics, namely erythromycin, ciprofloxacin, gentamicin, and chloramphenicol. Notably, most of these isolates (n = 52) presented an unusual erythromycin-susceptibility/clindamycin-resistance phenotype. WGS showed that these isolates lacked the *erm* and the *lnu* genes encoding resistance to macrolides and lincosamides, respectively, but carried the *vgaA*_*LC*_ gene encoding resistance to lincosamides, which is here firstly identified in *S*. *aureus* ST398.

**Conclusion:**

After two years the ban of colistin and amoxicillin as feed additives had no significant impact on the MRSA nasal carriage rates. Nevertheless, the MRSA strains circulating in those farms showed resistance to a lower number of antibiotic classes.

## Introduction

There is increasing concern about the use of antibiotics in food-producing animals that may lead to elevated resistance rates, and therefore ultimately impact the treatment of human infections. By consequence, several countries in the European Union have made efforts to reduce the use of antibiotics in livestock, in particular limiting their use as growth promoters and prophylaxis in healthy animals.

Livestock-associated methicillin-resistant *Staphylococcus aureus* (LA-MRSA) have been widely reported as nasal colonizers of pigs in many geographical areas [1]. Porcine MRSA in Europe and the United States mainly belong to clonal complex (CC)398 [1]. In 2016, we evaluated the occurrence of MRSA isolates in two pig farms in Portugal (farms A and B) that supplemented the feed regimen of the animals with colistin and amoxicillin. Very high rates of MRSA (99%) were found in both farms, and all strains belonged to ST398 [2]. Since then, as a consequence of the Portuguese national action plan for the reduction of the use of antibiotics in animals [3], several farms stopped feeding the pigs with colistin-supplemented regimens, including farms A and B, and some farms completely abolished the routine use of any antibiotic prophylaxis.

Although there is clear evidence about the relationship between a high antimicrobial usage in pig farms and the increased rates of Gram-negative resistant bacteria in their digestive tract [4–7], there is no study evaluating the impact of the use of antimicrobials on the nasal carriage of multidrug-resistant MRSA in pigs.

The aim of the present study was to evaluate the impact of the ban of colistin and amoxicillin from the feed regimens of healthy pigs on MRSA carriage rates, MRSA clonal types and antimicrobial resistance, compared to the isolates obtained in 2016 from animals receiving both antibiotics.

## Materials and methods

### Farms and study design

Two independent Portuguese pig farms (farms B and C), all located in the Alentejo region, were included in the study. All pigs are born in these farms and further delivered to slaughterhouses. The two farms used amoxicillin (0.5%), colistin (0.5%), and zinc oxide (0.15%) in the feed regimen of all animals until 2016. Since then, farm B banned colistin from the feed regimen, maintaining amoxicillin (0.5%) and zinc oxide (0.15%), while farm C did not use either antibiotic, keeping zinc oxide (0.15%) as a feed supplement for the prevention of gastrointestinal diseases. No other feed additives were included in the regular feed regimen at any time. However, the two farms administrated tetracycline in the feed regimens of all animals whenever more than 10% of the pigs developed a gastrointestinal infection.

**Fig 1:**
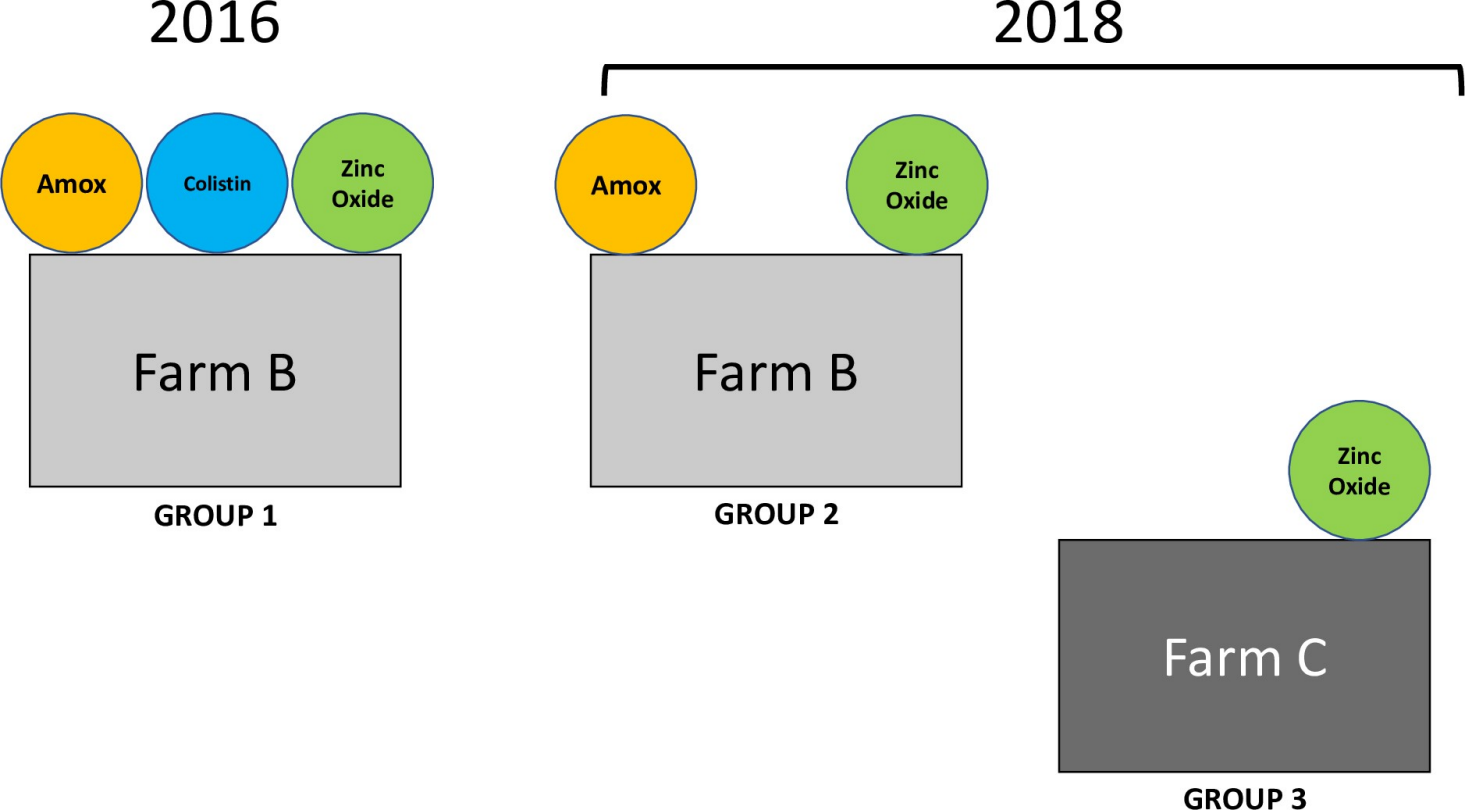
Diagram representing the three groups of pigs, considering the sampling period, the farm, and the feed regimen (amoxicillin, colistin, and zinc oxide).

A total of 154 piglets, aged 10–12 weeks, were randomly selected from different stockyards in each farm and nasally swabbed for MRSA colonization. Fifty-one pigs from farm B were swabbed in 2016 (group 1) and 103 pigs were swabbed in 2018 (52 pigs from farm B [group 2] and 51 pigs from farm C [group 3])–Fig 1. Unfortunately, we could not obtain samples from farm C in 2016. MRSA isolates obtained from group 1 were isolated and characterized in our previous study [2].

### Ethics statement

The protocol was approved by the Research Board of Escola Superior de Saúde da Cruz Vermelha Portuguesa.

### Sampling and MRSA identification

Samples were taken by swabbing a single nasal cavity of each animal with a sterile cotton swab, which was stored in Stuart transport medium. After overnight enrichment growth at 37ºC in Mueller-Hinton broth (Becton, Dickinson & Co, New Jersey, USA), the overnight samples were inoculated on Tryptic Soy Agar (TSA) (Becton, Dickinson & Co, New Jersey, USA) and Chromagar MRSA (ChromAgar, Paris, France). MRSA was confirmed by PCR amplification of the *spa* gene for species identification, and the detection of the *mecA* gene [8, 9].

### Molecular typing

The isolates were characterized by a combination of three typing methods. *Spa* typing was performed as previously described [9] and *spa* types were assigned through the Ridom web server (http://spaserver.ridom.de). Multilocus sequence typing (MLST) was performed as previously described[10] and the allelic profiles and sequence types (ST) were defined using the MLST online database (https://pubmlst.org/saureus/). Staphylococcal cassette chromosome *mec* (SCC*mec*) was characterized by multiplex PCR [11].

### Susceptibility testing

Antimicrobial susceptibility testing was performed by disk diffusion, according to the European Committee on Antimicrobial Susceptibility Testing (http://www.eucast.org), for the following antibiotics: cefoxitin (FOX), ciprofloxacin (CIP), chloramphenicol (CHL), clindamycin (CLIN), erythromycin (ERY), fusidic acid (FUS), gentamicin (GEN), linezolid (LZD), mupirocin (MUP), penicillin (PEN), quinupristin-dalfopristin (QD), rifampin (RIF), tetracycline (TET), and trimethoprim-sulfamethoxazole (SXT). Vancomycin (VAN) resistance was tested by E-test.

### Whole genome sequencing

Whole genomic DNA of six MRSA isolates belonging to the two major *spa* types, t011 (n = 3) and t108 (n = 3), was extracted with the Sigma-Aldrich GenElute^™^ Bacterial Genomic DNA Kit. Genomic libraries were assessed using the NexteraXT library preparation kit (Illumina Inc., San Diego, CA) and sequencing was performed using the Illumina MiniSeq system with 150-bp paired-end reads and a coverage of 50X. Generated FastQ data were compiled and analyzed using the CLC genomic workbench 7.5.1 (CLC bio, Aarthus, Denmark). Reads were de novo assembled with automatic bubble and word size and contigs were generated using the mapping mode “map reads back to contigs” with a minimum contig length of 800 nucleotides. Antimicrobial resistance genes were identified using the ResFinder database [12] and the Comprehensive Antibiotic Resistance Database (CARD) platform [13]. Since all pigs received zinc oxide in their feed regimen, the presence of the *czrC* gene encoding resistance to zinc and cadmium [14] was evaluated by a BLAST (Basic Local Alignment Search Tool) analysis over the GenBank database.

### Detection of resistance genes by PCR

Resistance genes detected by WGS on representative isolates, namely genes *fexA*, *dfrG*, *aac(6')-Ie-aph(2'')-Ia*, *ermA*, *ermC*, and *ermT* have been additionally confirmed by PCR [15–17].

### Statistical analysis

Chi-squared or Fisher’s exact test were used to determine the differences between the MRSA prevalence and resistance to the different antibiotics among isolates recovered from the three groups of pigs with different antibiotic feed regimens. *P*-values <0.01 were considered statistically significant.

## Results

### MRSA prevalence

Overall, 96% (99 out of 103) of the piglets swabbed in 2018 (farm B n = 48/52 and farm C n = 51/51) were nasally colonized with MRSA. This rate was similar to the one found in 2016 (99%) [2]. No differences in MRSA prevalence were observed regarding the three groups of pigs under different antibiotic feed regimens (p<0.01).

### Molecular characterization of MRSA

All isolates (n = 157) belonged to ST398, independently of the farm, sampling period, and antibiotic administration in the feed regimen. Most of the isolates harbored SCC*mec* type V (n = 146/157; 97%), while 3% were SCC*mec* non-typeable by the multiplex strategy (amplification of *mecA* was obtained only). Three *spa* types were detected within the whole collection (2016 and 2018), namely t011 (n = 108; 70%), t108 (n = 45; 29%), and a novel type t18272 (n = 4; 3%)–Table 1. The two major types, t011 and t108, were present in the four groups of isolates, while t18272 was exclusively found in group 2. Interestingly, t011 was the predominant clone in all groups with the exception of group 1 in which 62% of the isolates corresponded to t108.

**Table 1:** Distribution of *spa* types of the 157 ST398-MRSA isolates from the three groups of pigs.

*spa* type	Group 1 [2] [Farm B; 2016; AMX+COL]*	Group 2 [Farm B; 2018; AMX]*	Group 3 [Farm C; 2018; none]*	Total
t011	22 (38%)	**38 (79%)**	**48 (94%)**	**108 (70%)**
t108	**36 (62%)**	6 (13%)	3 (6%)	45 (29%)
t18272		4 (8%)		4 (3%)
Total	58	48	51	157

*Antibiotics included in the feed regimen. AMX–Amoxicillin; COL–Colistin.

The prevalent *spa* type in each group is displayed in bold.

Percentages referred to the total number of isolates in each group.

### Antimicrobial susceptibility

None of the isolates showed decreased susceptibility to fusidic acid, linezolid, rifampicin, mupirocin, and vancomycin, while all isolates were resistant to cefoxitin and tetracycline, and 99% were resistant to clindamycin and QD.

Analysis of the antimicrobial susceptibility profiles of the MRSA isolates collected in the three groups, showed striking differences (Table 2). Overall, in group 1 (receiving colistin and amoxicillin in the feed regimen in 2016), ciprofloxacin was the single antibiotic to which more than 50% of the isolates were susceptible, while in group 2 (receiving amoxicillin only) the large majority of the isolates was susceptible to gentamicin and chloramphenicol, and in group 3 (pigs not receiving antibiotics) the majority of the isolates remained susceptible to four antibiotics (erythromycin, ciprofloxacin, gentamicin, and chloramphenicol).

**Table 2:** Antibiotic resistance of the 157 ST398-MRSA isolates from the three groups of pigs.

	Total	Group 1 [2] [Farm B; 2016; AMX+COL]^a^	Group 2 [Farm B; 2018; AMX]^a^	Group 3 [Farm C; 2018; none]^a^
**Total collection**	**157**	**58**	**48**	**51**
FOX	**157 (100%)**	**58 (100%)**	**48 (100%)**	**51 (100%)**
ERY	**106 (68%)**	58 (100%)*	46 (96%)*	2 (4%)*
CLIN	**155 (99%)**	**58 (100%)**	**48 (100%)**	**49 (96%)**
CIP	67 (43%)	22 (38%)	42 (88%)*	3 (6%)*
TET	**157 (100%)**	**58 (100%)**	**48 (100%)**	**51 (100%)**
SXT	**114 (73%)**	31 (53%)*	44 (92%)*	**39 (76%)**
GEN	54 (34%)	**29 (50%)**	2 (4%)*	23 (45%)
QD	**156 (99%)**	**58 (100%)**	**48 (100%)**	**50 (98%)**
CHL	52 (33%)	51 (88%)*	1 (2%)*	0*
***spa* t011**	**108**	**22**	**38**	**48**
FOX	**108 (100%)**	**22 (100%)**	**38 (100%)**	**48 (100%)**
ERY	**60 (56%)**	22 (100%)*	36 (95%)*	2 (4%)*
CLIN	**106 (98%)**	**22 (100%)**	**38 (100%)**	**46 (96%)**
CIP	**63 (58%)**	22 (100%)*	38 (100%)*	3 (6%)*
TET	**108 (100%)**	**22 (100%)**	**38 (100%)**	**48 (100%)**
SXT	**99 (92%)**	**22 (100%)**	**38 (100%)**	39 (81%)*
GEN	22 (20%)	0*	0*	22 (46%)
QD	**107 (99%)**	**22 (100%)**	**38 (100%)**	**47 (98%)**
CHL	15 (14%)	15 (68%)*	0*	0*
***spa* t108**	**45**	**36**	**6**	**3**
FOX	**45 (100%)**	**36 (100%)**	**6 (100%)**	**3 (100%)**
ERY	**41 (91%)**	36 (100%)*	**5 (83%)**	0*
CLIN	**45 (100%)**	**36 (100%)**	**6 (100%)**	**3 (100%)**
CIP	0 (0%)	0*	0*	0*
TET	**45 (100%)**	**36 (100%)**	**6 (100%)**	**3 (100%)**
SXT	11 (24%)	9 (25%)	2 (33%)	0*
GEN	**32 (71%)**	29 (81%)*	2 (33%)	1 (33%)
QD	**45 (100%)**	36 (100%)*	**6 (100%)**	**3 (100%)**
CHL	**37 (82%)**	36 (100%)*	1 (17%)*	0*

FOX–Cefoxitin; ERY–Erythromycin; CLIN–Clindamycin; CIP–Ciprofloxacin; TET–Tetracycline; SXT–Trimethoprim-sulfamethoxazole; GEN–Gentamicin; QD–Quinupristin-dalfopristin; CHL–Chloramphenicol.

^a^Antibiotics included in the feed regimen. AMX–Amoxicillin; COL–Colistin.

*Significant difference (p<0.01).

Numbers in bold indicate that ≥50% of the isolates are resistant to the antibiotic.

### Antimicrobial susceptibility by *spa* type

By comparing the antimicrobial susceptibility profiles of all isolates belonging to each of the two major *spa* types (Table 2), resistance to ciprofloxacin and SXT was significantly higher among t011 isolates compared to t108 isolates (58% vs 0%; p<0.01 and 92% vs 24%; p<0.001, respectively), while resistance to erythromycin, gentamicin and chloramphenicol was lower in the former group (56% vs 91%, p<0.001; 20% vs 71%, p<0.01; and 14% vs 82%, p<0.001, respectively).

By comparing t011 isolates recovered from farm B pigs that received colistin and amoxicillin in 2016 (group 1) and isolates from the same farm, from pigs receiving only amoxicillin two years later (group 2), a difference in chloramphenicol susceptibility was demonstrated, with isolates from group 2 being susceptible while those of group 1 being resistant. Similarly, most of the t108 isolates from group 2 showed higher rates of susceptibility to gentamicin and chloramphenicol compared to those of group 1 (Table 2).

Likewise, the large majority of the isolates recovered from pigs that did not receive any antibiotic in the feed regimen (group 3), for both *spa* types, remained susceptible to erythromycin, ciprofloxacin, gentamicin, and chloramphenicol, and also to SXT for *spa* t108 (Table 2).

### Whole genome sequencing

**Fig 2:**
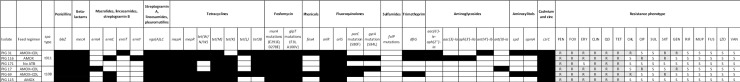
Resistance phenotype versus genotype obtained by whole genome sequencing for six ST398-MRSA representative isolates from pigs receiving different antibiotic feed regimens (amoxicillin + colistin, amoxicillin only, and no antibiotics). Black squares indicate presence of gene.

To gain insights into the antimicrobial resistance genotypes that may explain the different phenotypes observed among the different groups of isolates, WGS was performed for six isolates representatives of the three groups (Fig 2).

Sequence analysis followed by PCR showed that, independently of the *spa* type, resistance to chloramphenicol was due to the presence of the phenicol exporter encoding gene *fexA*, resistance to SXT to the dihydrofolate reductase encoding gene *dfrG*. Isolates resistant to aminoglycosides carried at least one gene encoding aminoglycoside modifying enzymes, namely *aac(6')-Ie-aph(2'')-Ia*, *ant(4')-Ib*, or *ant(9)-Ia*. Isolates resistant to ciprofloxacin had mutations in ParC (S80F) and GyrA (S84L). Isolates with only the S80F substitution in ParC remained susceptible to ciprofloxacin, which is in agreement with a previous study showing that only the S80Y substitution in ParC may confer moderate level of resistance to fluoroquinolones [18] if no mutation in GyrA was associated.

Interestingly, the *spd* and *apmA* inactivation genes conferring resistance to aminocylitols were absent in isolates from groups 2 and 3, while the *czrC* gene encoding a heavy metal translocating P-type ATPase conferring resistance to zinc and cadmium was present in the genome of the six sequenced isolates. Moreover, all sequenced isolates carried not only the *mepA* and *mepR* genes coding for multidrug efflux pumps, but multiple (≥3) *tet* genes encoding multidrug efflux pumps of the Major Facilitator Superfamily as well, explaining the tetracycline resistance phenotype.

A total of 52 isolates (33% of the whole collection) presented an unusual erythromycin-susceptible and clindamycin-resistant phenotype. These isolates belonged mainly to clonal lineage ST398-t011-SCC*mec*V (n = 48; 92%) and 47 (90%) belonged to group 3. Sequencing of a representative isolate showing susceptibility to macrolides and resistance to lincosamides (PIG171) showed that it lacked the *erm* genes and carried the *vgaA*_*LC*_ gene that encodes resistance to lincosamides [19].

## Discussion

To our knowledge, this is the first study analyzing the impact overtime of different antibiotic feed regimens, namely the ban of colistin and amoxicillin, on the MRSA nasal carriage among healthy pigs. No difference in the MRSA prevalence was observed when considering feed regimens containing either colistin and amoxicillin, amoxicillin only, or no antibiotic.

All isolates belonged to ST398, mainly associated with SCC*mec* V and *spa* types t011 and t108. This suggests the reduction and even elimination of antibiotics in the feed regimen of pigs, namely amoxicillin, does neither affect the rate of nasal MRSA carriage nor the MRSA clonal type. Several reports from Europe identified t011, t034 and t108 as the major *spa* types in CC398-MRSA from animals, retail meat and human isolates [20–22]. However, t034 was not found in our study and has actually never been reported in Portugal.

In the present study, all MRSA isolates were resistant to tetracycline and carried the *mepA* and *mepR* genes as well as multiple *tet* genes. Tetracyclines are the most frequently used antibiotics among pigs in Portugal (83.9 mg/PCU in 2016), followed by penicillins (46.3 mg/PCU), macrolides (21.5 mg/PCU), and colistin (13.5 mg/PCU) [23]. Noticeably, although tetracycline was not included daily as a feed additive, both farms administrated this antibiotic in the feed regimens of all animals whenever more than 10% of the pigs developed a gastrointestinal infection, which may contribute to the high rate of resistance to this antibiotic.

Also, all isolates harbored the *czrC* gene, which is frequently localized together with *mecA* on SCC*mec* elements from LA-MRSA, in particular SCC*mec* type V [24]. The fact that zinc oxide was given as a feed supplement constituted a selective pressure for acquisition of β-lactam resistance, and therefore selection of MRSA, despite the lack of β-lactam selective pressure. Future studies in farms that will bann the use of zinc oxide in the feed regimen will be of interest to confirm this phenomenon. Moreover, it has been shown that the presence of both *tet(K)* and *tet(M)* confers a fitness advantage to LA-MRSA CC398, which associated to *czrC* might drive the expansion of this clone [25].

In our collection, MRSA isolates recovered from pigs receiving no antibiotic in the feed regimen showed susceptibility to a higher number of antimicrobial agents compared to isolates from pigs receiving colistin and/or amoxicillin, suggesting that a lower antimicrobial exposure correlates with a lower rate of antibiotic resistance among MRSA colonizing the anterior nares of healthy pigs. This is in agreement with previous studies that showed that any form of antimicrobial exposure in swine, including different modes of administration, actually increases the prevalence of antibiotic-resistant bacteria in their gut [5, 6]. Of note, many of the antibiotic resistance genes found among isolates recovered from pigs receiving antibiotics in the feed regimen and absent in isolates recovered from pigs not receiving antibiotics are plasmid-encoded, namely *ermT*, *tetL*, *fexA*, *spd*, *apmA*, and *dfrG* [15, 26]. Therefore, the absence of antibiotic selective pressure might have driven the loss of these genetic elements over time, that in most cases are of small size (<15 kb). However, given that in some cases the observed differences in antibiotic susceptibility are outside of the classes of drugs no longer administered, these changes might be potentially just happenstance.

Another important finding from this surveillance study was the detection of a high proportion of MRSA isolates presenting erythromycin susceptibility and clindamycin resistance (25%), mostly in isolates recovered from pigs receiving no antibiotic in the feed regimen (90%). Previous studies have found this uncommon phenotype among MRSA-ST398 swine isolates associated with the *lnu(A)* or *lnu(B)* genes and this phenotype seems to be related to *S*. *aureus* animal-associated clonal lineages [27, 28]. Our isolates did not carry any of the *lnu* genes but instead harbored the *vgaA*_*LC*_ gene that encodes resistance to lincosamides. This variant of the *vgaA* gene has substrate specificity towards lincosamides and has been previously found in clinical isolates of *Staphylococcus haemolyticus* resistant to lincomycin/clindamycin but susceptible to erythromycin, for which no relevant lincosamide resistance gene was found [19]. The *vgaA*_*LC*_ gene has been previously reported in two *S*. *aureus* human clinical isolates responsible for skin and soft tissue infection that showed the same erythromycin susceptibility and clindamycin resistance phenotype [29]. Moreover, *vgaA*_*LC*_ was detected in the genome of a single swine LA-MRSA ST5 isolate recovered in the United States [30, 31], but to our knowledge, this is the first identification of the *vgaA*_*LC*_ gene in the widely widespread LA-MRSA lineage ST398.

In summary, MRSA currently colonizing the nares of healthy pigs in Portugal belong to ST398-V, mainly associated with *spa* types t011 and t108. A considerable proportion of MRSA-ST398-t011 isolates presented the unusual phenotype macrolide-susceptibility/lincosamide-resistance associated to the presence of the *vgaA*_*LC*_ gene. Pigs receiving less antibiotics as feed additives but still receiving zinc oxide maintained high MRSA nasal carriage rates, which was likely related to this heavy metal selective pressure. However, those MRSA isolates colonizing the pigs were resistant to less classes of antibiotics.
